# Road to Tokyo 2020 Olympic Games: Training Characteristics of a World Class Male Triathlete

**DOI:** 10.3389/fphys.2022.835705

**Published:** 2022-04-20

**Authors:** Roberto Cejuela, Sergio Sellés-Pérez

**Affiliations:** Physical Education and Sports, Faculty of Education, University of Alicante, Alicante, Spain

**Keywords:** triathlon, training load, ECOS, training periodization, endurance training

## Abstract

There is a growing interest in the scientific literature for reporting top-class endurance athletes training programs. This case study reports on the training program of a world-class male triathlete preparing to compete in the Tokyo 2020 Olympic Games. A macrocycle of 43 weeks is presented. The triathlete performed 14.74 ± 3.01 h of weekly endurance training volume. Training intensity distribution (TID) was 81.93% ± 6.74%/7.16% ± 2.03%/10.91% ± 6.90% for zones 1 (low intensity, <VT1), 2 (moderate intensity, VT1-VT2) and 3 (high intensity, >VT2) respectively. Pyramidal TID model is observed during the initial stages of the periodization and Polarized TID model is observed at the end of the macrocycle. The triathlete’s peak ⩒O_2_ was increased by 20% on cycling and by 14% on running. Peak power was increased by 3.13% on cycling test and peak speed by 9.71% on running test. Finally, the triathlete placed 12th in Olympic distance and 10th in Mixed Relay in Tokyo 2020 Olympic games.

## Introduction

The summer Olympic Games are organized every 4 years with a great socioeconomic impact, since they have an audience of more than three billion viewers around the world (Yan, 2020). Triathlon is an endurance sport that combines swimming, road cycling and distance running performed in that order (ITU, 2021). This sport has been part of the Olympic program since Sydney 2000 with the Olympic distance discipline (1.5 km swimming, 40 km cycling and 10 km running). Tokyo 2020 has been the first edition to include the mixed relay format, where the four members of each participating team (i.e, two women and two men) each complete a full super sprint distance triathlon (0.3 km swimming, 8 km cycling and 2 km running). 55 triathletes participated in the Olympic distance of the Tokyo 2020 Olympic Games, previously qualified according to different criteria 2 years before the event. A maximum of three triathletes from the same country can take part in the individual event in both male and female categories. Regarding to the mixed relay race, a total of 18 teams took part in the premiere of this modality in the Olympic Games (ITU, 2021). Besides, Tokyo 2020 Olympic Games were considered particularly hard due to the extreme weather conditions. Meteorological data indicated that weather condition were both hot (26–28°C) and humid (87–89%) during the triathlon races. Such conditions affect the endurance performance and increase the risk of exertional heat illness (Racinais et al., 2015a).

There is a growing interest in the scientific literature for reporting top-class endurance athletes’ successful programs (Ingham et al., 2012; Tnønessen et al., 2014; Rønnestad and Hansen, 2018; Tjelta, 2019; Haugen et al., 2021; Kenneally et al., 2021). Variables such as a total training volume, exercise intensity and training intensity distribution (TID) have been commonly analyzed in this kind of researches. TID is defined as the time of the exercise that an athlete spends at the three different zones of training intensity (Stöggl and Sperlich, 2014): zone 1, at or below the first ventilatory threshold (<VT1); zone 2, between first and second ventilatory threshold (VT1-VT2); zone 3, at or beyond the second ventilatory threshold (>VT2) (Skinner and McLellan, 1980). Polarized model, which is based on a high percentage of time at zone 1 and greater percentages at zone 3 than at zone 2, has been presented as the optimal model of TID to enhance the performance of endurance athletes (Seiler and Kjerland, 2006; Neal et al., 2013; Stöggl and Sperlich, 2014). However, not all findings on polarized TID point to its superiority (Treff et al., 2017). Besides, other TID models as threshold or pyramidal, which accumulate a greater percentage of time at zone 2 than polarized model, has also been presented as effective (Plews and Laursen, 2017; Selles-Perez et al., 2019; González-Ravé et al., 2021). On the other hand, fewer studies analyze training load using specific training load quantification methods for endurance sports (Esteve-Lanao et al., 2017; Selles-Perez et al., 2019).

Physiological profiles of elite endurance athletes have been also reported in several research (Ingham et al., 2012; Bell et al., 2017). VO_2max_, ventilatory thresholds, as well as power or speed at which these variables occur, these have been common parameters used by coaches to assess fitness levels and prescribe training plans. Even more, it is particularly interesting to describe the dynamics of these variables along the macrocycle (Legaz Arrese et al., 2005; Legaz and Eston, 2005; Sellés-Pérez et al., 2019).

There are limited reports about training characteristics and physiological performance of top-level triathletes (Millet and Vleck, 2000; Millet et al., 2003). Mujika (2014) described training characteristics of a female triathlete in her preparation to the London 2012 Olympic Games. Later, this author presented the physiological profile and the training plan for a world-champion male paratriathlete (Mujika et al., 2015). However, to the best of our knowledge, no case report has been published of world class male triathlete preparing for the Olympic Games. Thus, the aim of this case study is to describe the training characteristics and the physiological profile of a world-class male triathlete who participated in both the Olympic distance event and the mixed relay event at the Tokyo 2020 Olympic Games.

## Materials and Methods

### Characteristics of the Triathlete

This case study received the authorization from the Alicante University Ethics Committee (UA-2017-04-11 expedient). Written consent was obtained from a 29 years-old male triathlete who has competed in world triathlon series since 2012 obtaining 20 podiums and four victories in International Triathlon Union (ITU) races. He finished in fourth position of the World Triathlon Series (WTS) ranking in 2019, obtaining the qualification for the Tokyo 2020 Olympic Games. He also competed in Rio 2016, where he placed 18th.

### Physiological Testing, Anthropometric Measures and Training Zone Settings

Swimming test were performed on a 25 m homologated swimming pool through an incremental multistage test consisting of seven repetitions of 200 m every 5 min (Sweetenham and Atkinson, 2003; Muñoz et al., 2014). The first repetition of the protocol was 20 s slower than his personal best in 200 m registered the previous week in a training session and then every repetition was 4 s faster until the sixth, and for the seventh repetition the triathlete was told to perform to swim as fast as possible. Blood lactate (bLA; mmol/l) samples from the ear lobe were analyzed with a portable lactate analyzer (Lactate Scout® 4, EKF Diagnostics, Germany). The criteria to determine training zones were follows in swimming: a blood lactate 0.5 mMol/L increase for first lactate threshold (LT_1_), >1 mmol/l increase for second lactate threshold (LT_2_) and 8–9 mmol/l for maximal aerobic speed (MAS) (Beneke, 2003; Jamnick et al., 2020).

Incremental tests of volitional exhaustion were used to determine training zones in cycling and running. A ramp protocol was used for cycling on a roller (Cycleops® The Hammer, United States) starting at 150 W and increasing 5 W each 12 s (Muñoz et al., 2014). The triathlete used his own bike (BH®, Aerolight, Spain) and his power meter (ROTOR®, 2inpower Road, Spain) to perform the test. The cycling tests were performed in the same room with the same temperature (20°C). The running test was performed on a 400 m homologated track (University of Alicante facilities). The triathlete started at 13.9 km/h and increased 0.3 km/h every 200 m (Brue, 1985). The triathlete uses the same running shoes in both test (ASICS®, Metaspeed Edge, Japan). Weather conditions were a temperature of 19°C and a wind of 7.5 km/h for the first running test, while they were a temperature of 15°C and a wind of 5.4 km/h for the second test.

Running and cycling tests were conducted using a portable gas-exchange analyzer (Cosmed® K4b 2, Italy). The following variables were measured during the test: oxygen uptake (⩒O_2_); pulmonary ventilation (⩒E); ventilatory equivalent for oxygen/⩒E/⩒O2); ventilatory equivalent for carbon dioxide (⩒E/⩒CO_2_); and end-tidal partial pressure of oxygen (P_ET_O_2_) and carbon dioxide (P_ET_CO_2_). Maximal oxygen uptake (⩒O_2max_) was recorded as the highest ⩒O_2_ value obtained for any continuous 1 min period. The first ventilatory threshold (VT1) was determined using the criteria of an increase in both ⩒E/⩒O_2_ and P_ET_O_2_ with no increase in ⩒E/⩒CO_2_, whereas the second ventilatory threshold (VT2) was determined using the criteria of an increase in both ⩒E/VO_2_ and ⩒E/⩒CO_2_ and a decrease in P_ET_CO_2_. Two independent observers identified VT1 and VT2. Heart rate (HR) was continuously monitored during the test using a heart rate meter (SUUNTO®, Spartan, Finland). Later, a range of HR and power or velocity for each training zone was established. Eight training zones were calculated to be more precise prescribing the intensity of the training sessions. These training zones reported both internal load (HR) and external load (speed or power) data (Cejuela and Esteve-Lanao, 2011, 2020). Besides, a RPE scale (1–10) was related to these training zones. Additionally, the Skinner and McLellan triphasic model with three training zones was followed to present TID and training load distribution (TLD) data.

Anthropometric measurements were performed following standard protocols adopted by the international society for the Advancement on Kinanthropometry (ISAK) (Ross and Marfell-Jones, 1991) by the same researcher with ISAK certification level 3. All measurements were taken on the day of the cycling test under basal conditions, in the same room with the same temperature (22°C). Height and body mass were measured on portable set scales (models 213 and 707, Seca®, Deutschland) to the nearest 0.1 cm and 0.01 kg, respectively. The thickness of six skinfolds (subscapular, triceps, supraspinale, abdominal, front thigh and medial calf) were measured using a caliper calibrated to the nearest 0.2 mm (Holtain®, United Kingdom). Four girths (relaxed arm, flexed arm, thigh and calf) were performed using flexible anthropometric steel tape (Holtain®, United Kingdom). The sum of skinfolds was calculated, as well as muscular mass was determined using the method of Lee et al. (2000).

### Control of the Training Load

Cycling power data were measured using a power meter located on the crank (ROTOR®, 2inpower Road, Spain). The triathlete recorded all cycling and running training sessions with his HR monitor (SUUNTO®, Spartan, Finland) and after the data were uploaded to a specific data analysis software (TrainingPeaks®,WKO5, United States). HR and RPE were used mainly for low intensity workouts in running and cycling. Speed and power were also used to control moderate and high intensity workouts in running and cycling. RPE and the medium pace for 100 m were used to control swimming workouts, considering the different training zones obtained in the swimming lactate test. Besides, the triathlete was filling personal training logs with the information recorded regarding the amount of time spent in each training zone and with other subjective information such as hours of sleep and with the subjective load scale (ECSs in Spanish) from 0 to 5 (Cejuela and Esteve-Lanao, 2011; Cejuela and Esteve-Lanao, 2020). Execution speed was controlled in strength training using a linear encoder (VITRUVE®, Spain). Most of the training workouts was supervised by the coach of the triathlete (RC) or an assistant coach (SS or AT).

The objective load scale (ECOs in Spanish) training load quantification method was applied (Cejuela and Esteve-Lanao, 2011; Cejuela and Esteve-Lanao, 2020). Briefly, the ECOs were calculated by multiplying the time (minutes) that the triathlete spends in every training zone (1–8) during the workout by a scoring value between 1 and 50 (depending on the training zone) and by a specific factor of 1.0, 0.75 or 0.5 for running, swimming or cycling respectively. This methodology seems the most appropriate for triathlon because it compares different endurance activities, attending the different degrees of muscle damage, energy cost, effort densities, and differences at the ability of maintaining technique in the three segments (Cejuela and Esteve-Lanao, 2011; Cejuela and Esteve-Lanao, 2020). A specific software (All in your mind Training system®, Mexico) was used to calculate the ECOs throughout the training plan.

### Main Characteristics of the Training Period

The triathlete trained with the same methodology in the 2019 and 2020 seasons. The goal was competing in the WTS in 2019, where he ranked fourth. Due to COVID-19 there were no WTS in 2020. This season he was Spanish championship.

The training period for the Tokyo Olympic Games consisted out of 43 weeks, which were grouped in a total of 14 mesocycles. The main part of the training was developed in Alicante (Spain) or in Talavera de la Reina (Spain). Besides, two training camps of 4 weeks were performed in the high-altitude performance center of Sierra Nevada (2,340 m altitude, Spain). Simulated altitude sessions (iAltitude®, Spain) were performed at three times of the season (daily from week 8–10, from week 14–16 and from week 36–40). The duration of the sessions was from 45 to 90 min and the range of altitude exposures was from 3,500 to 6,000 m. The first physiological tests took place at week 6 (swimming) and 7 (cycling and running test). The second physiological tests were performed at week 24. A traditional periodization was used in the first part of the season. The general preparatory period was performed from week 1–14. These weeks were characterized by an increasing of training volume progressively, being the most part of the training sessions around VT1. The specific preparatory period was performed from week 15–30, including the two training camps in high-altitude performance center. The first one was from week 18–21 and the second one was from week 27–30. The main goal of training period consisted in develop higher training zones, nearer to race intensity (VO_2max_ and VT2). Usually, transitions trainings were performed twice weekly with intensities nearer to the competition (around VT2). The triathlete also took part in two competitions during this period in order to training, one sprint duathlon at week 24 (1st) and one Olympic triathlon at week 28 (1st). The first competitive period lasted 5 weeks (from week 31 to week 35) and included two Olympic-distance triathlon world series (WTS). The first one in Yokohama (week 32) where the triathlete ranked at 14th position and the second one in Leeds (week 35) where the triathlete ranked at 4th position. A Mixed relay Olympic qualification event were also performed at week 33 at Lisbon, where the team of Spain ranked at 6th. A tapering period took place during this competitive period. The aim goal of these weeks was reduced training load to promote the supercompensation of the triathlete and improve the recovery. This was mainly done through the training volume reduction. High intensity training sessions were maintained during this period. A 5 weeks training block (from week 36 to week 40) of specific preparation to Tokyo Olympic Games was conducted after these three competitions. Training load was increased again during this period. Tapering period to the Olympic games was performed during weeks 41 and 42, through the decrease of the training load in an exponential manner, in order to promote supercompensation. Besides, these weeks included the adaptation period to the Tokyo jet lag. Finally, the triathlete competed in both the Olympic distance triathlon event and the mixed relay triathlon event at week 43.

The triathlete also performed strength training throughout his preparation to the Olympic games. As a general rule, two weekly strength training sessions were performed during the most part of the season. Multi-joint exercises, both upper and lower body, were performed by the triathlete. Training loads progressed from 55% to 75% of 1RM, performing from two to four sets per exercise. The rep range was from four to eight reps per set, always working away from muscle failure and with a loss of speed not greater than 15% within the set. Furthermore, complementary exercises were carried out three or 4 days a week, after the swimming training sessions. These exercises included hip, ankle and thoracic mobility, core training, Achilles tendon prevention exercises or exercises with elastic bands for the shoulder stability.

Polarization index was calculated to quantify the level of polarization every week (Treff et al., 2019). TLD, defined as the percentage of objective training load (ECOs) that the triathlete performs in zones 1, 2 and 3, was also calculated every week.

### Heat Adaptation Protocol

A heat adaptation protocol was carried out in order to minimize the impact on performance of the adversely expected weather conditions in Tokyo (high humidity and temperature). On the one hand, the triathlete performed a heat acclimation using the passive method of sauna from week 18 to the begin of the first competitive period in alternate weeks. The sauna session was performed at the end of the training day. The triathlete was exposed for 20–30 min to a sauna bath with a temperature of 70–80°C (Racinais et al., 2019). On the other hand, the weather of Alicante was used to do the acclimatization from week 34 to the Olympic Games. Thus, the triathlete was gradually exposed to training at intensities close to the competition in the middle hours of the day. These training sessions were performed with a temperature from 25 to 32°C and with a humidity from 70% to 80%. A specific non-invasive sensor to assess core body temperature (CORE®, green TEG, Switzerland) was used in this training sessions (Verdel et al., 2021). A special focus on hydration was done during this period. Thus, recovery drinks with sodium were used to compensate for the sweat losses, while maintaining the optimal requirements of carbohydrates and protein to optimize recovery (Racinais et al., 2015a). Finally, the triathlete spent 10 days in Tokyo before the first competition performing several training sessions to maintain the heat adaptation.

## Results

**FIGURE 1:**
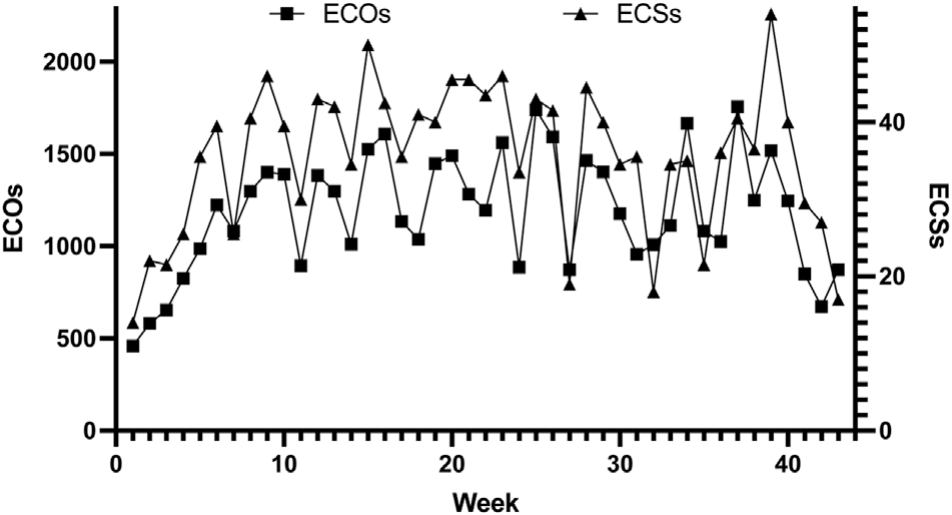
Weekly subjective training load (ECSs) related with the objective training load (ECOs).

Figure 1 shows the weekly subjective training load (ECS) reported by the triathlete and its relationship with the objective training load (ECOs). Peak of objective training load was performed at week 37 (1757 ECOs). Training load of the competitions was included in the summary. Peak of subjective training load was reported at week 39 (ECS), 1 month before to Tokyo Olympic Games. This subjective training load is related to one of the weeks with more objective training load (week 39, 1517 ECOs).

**FIGURE 2:**
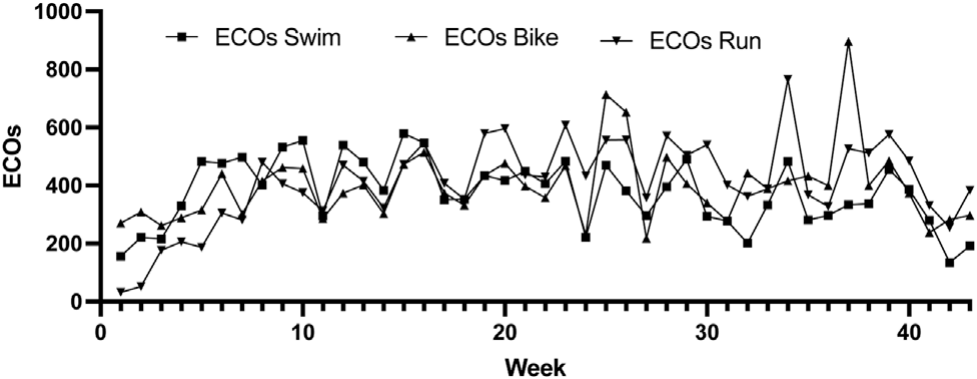
Weekly training load per discipline.

Figure 2 shows the weekly training load (ECOs) for swim, bike and run respectively. It is observed the peak training load for swimming at week 15 (579 ECOs), for cycling at week 37 (896 ECOs) and for running at week 34 (766 ECOs).

**FIGURE 3:**
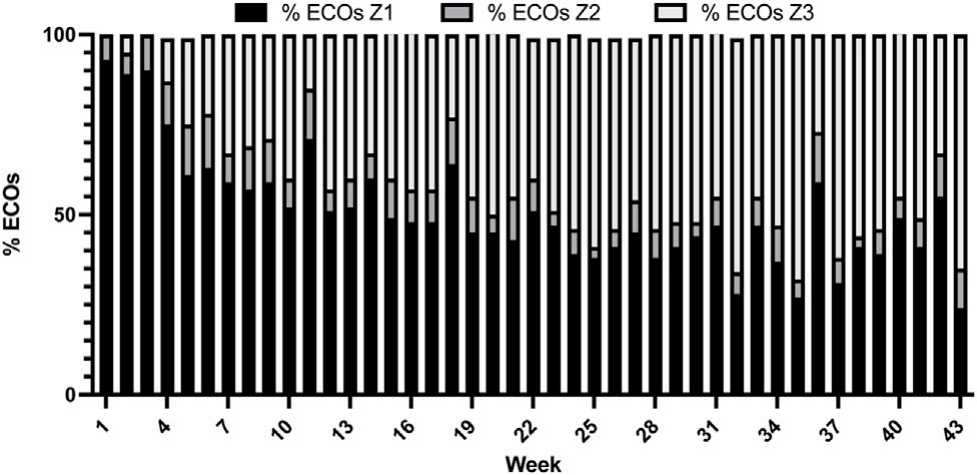
Weekly training load distribution (ECOs distributed at triphasic model).

Training intensity distribution, training volume, and polarization index of the weeks are presented in Table 1. After 4 weeks of low intensity training (>90% of training volume in zone 1) a pyramidal model is shown from week five to week 18, with the exception of week 12 (polarization index = 2.2). From 19 until the end of the training period a polarized training intensity distribution were carried out by the triathlete the majority of the weeks. Figure 3 shows the training load distribution. It is observed that 50.6% ± 15.5% of the training load was performed at zone 1 and 40.7% ± 16.8% was performed at zone 3.

**TABLE 1:** Weekly training volume and training intensity distribution (triphasic model).

Week	Volume (hours)	% Z1	% Z2	% Z3	P.I.	Week	Volume (hours)	% Z1	% Z2	% Z3	P.I.
1	10.4	95	5	0	0.0	23	18.3	83	4	13	2.4
2	11.6	95	5	0	0.0	24	13.6	78	6	16	2.3
3	12.3	93	7	0	0.0	25	20.1	71	3	26	2.8
4	12.1	92	7	1	1.1	26	18.7	79	7	14	2.2
5	13.3	86	9	5	1.7	27	13.4	82	8	10	2.0
6	17	84	12	4	1.4	28	17.9	78	6	16	2.3
7	12.9	89	6	5	1.9	29	14.1	80	6	14	2.3
8	15.9	83	11	6	1.7	30	12.5	78	7	15	2.2
9	17.9	85	9	6	1.8	31	11.6	82	7	11	2.1
10	16.2	85	7	8	2.0	32	10	75	5	20	2.5
11	13.7	89	9	2	1.3	33	15.4	77	7	16	2.2
12	16.4	86	5	9	2.2	34	17.3	71	11	18	2.1
13	16.1	84	7	9	2.0	35	9.6	66	7	27	2.4
14	13.9	88	6	6	1.9	36	16.6	82	10	8	1.8
15	17.9	83	9	8	1.9	37	17.1	77	7	16	2.2
16	18.5	84	8	8	1.9	38	15	75	2	22	2.7
17	13	84	8	8	1.9	39	17.6	77	7	16	2.2
18	15.2	86	9	5	1.7	40	15.3	85	5	10	2.2
19	17.5	84	6	10	2.1	41	10	75	8	17	2.2
20	18	84	5	11	2.3	42	10.1	82	9	9	1.9
21	16.6	82	8	10	2.0	43	7.3	64	10	26	2.2
22	16	85	7	8	2.0	Average	14.7 ± 3.0	81.9 ± 6.7	7.2 ± 2.0	10.9 ± 6.9	1.9 ± 0.6

Z, zone; P.I., polarization index.

Anthropometric measurements are presented at Table 2. A decrease in total body mass and sum of skinfolds are shown. However, few losses in muscular mass are reported. The change in physiological measures and performance during the season is observed at Table 3. The performance of the athlete was increased considerably in the three segments.

**TABLE 2:** Anthropometric measurements.

Variable	Anthropometry 1	Anthropometry 2
Body height (cm)	179.0	179.0
Body mass (kg)	68.8	66.3
∑ 6 Skinfolds	34.0	26.5
Muscular Mass (kg)	31.1	30.9

∑ 6 Skinfolds, Sum of six skinfolds; Muscular Mass (kg) (Lee et al., 2000).

**TABLE 3:** Change in physiological measures and performance during the season.

		LT1			LT2			MAS/Peak HR		
		Test 1	Test 2	% Change	Test 1	Test 2	% Change	Test 1	Test 2	% Change
**Swimming**	**Speed (m/s)**	1.40	1.42	+1.4%	1.49	1.51	+1.3%	1.56	1.61	+3.2%
	**Lac**	3.5	2.8	−20%	6.5	4.1	−36.9%	10.5	10	−4.8%
	**HR**	155	145	−6.5%	170	160	−5.9%	181	178	−1.7%
		**VT1**			**VT2**			**VO_2Max_/Peak power/Peak Speed/Peak HR**		
		**TEST 1**	**TEST 2**	**% Change**	**TEST 1**	**TEST 2**	**% Change**	**TEST 1**	**TEST 2**	**% Change**
**Cycling**	**P**	240	280	+16.7%	365	405	+11.0%	480	495	+3.1%
	**P/BM**	3.5	4.2	+22.0%	5.3	6.1	+16.0%	7.0	7.50	+7.8%
	**VO_2_**	44.2	57.1	+7.8%	61.3	72.2	+17.8%	70.5	84.0	+19.2%
	**HR**	140	140	0%	170	168	−1.2%	186	185	−0.5%
**Running**	**Speed (km/h)**	15.8	16.2	+2.5%	19.4	20.2	+4.1%	20.6	22.6	+9.7%
	VO_**2**_	43.2	54.8	+26.9%	57.6	69.5	+20.7%	72.0	81.8	+13.6%
	**HR**	152	153	+0.7%	172	173	+0.6%	190	191	+0.5%

LT, lactate threshold; P, Power (watts); P/BM, Power/Body Mass (w/kg); VO_2_, Oxygen uptake (ml/kg/min); Lac, Blood lactate (mmol/L); HR, Heart Rate (bpm); % Change, Percentage of change between test one and test two.

**FIGURE 4:**
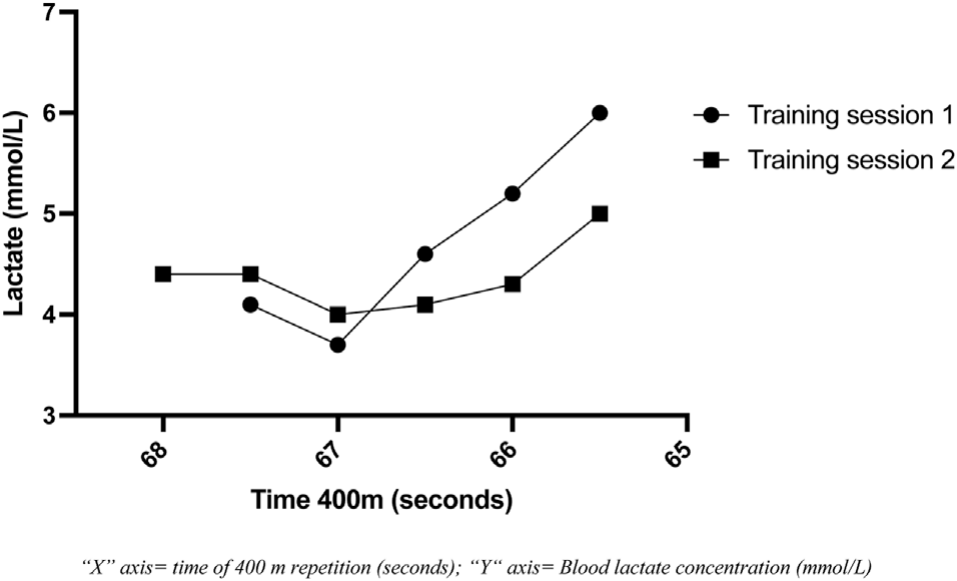
Blood lactate concentration of two running training sessions in heat condition.

Lactate blood concentration during two running training sessions in heat condition is presented in Figure 4. Training session 1 (week 36, 31°C and 68% of humidity) consisted in twenty repetitions of 400 m in a track (20 × 400). Training session 2 (week 40, 27°C and 74% of humidity) consisted in twenty-four repetitions of 400 m in the same track (24 × 400). The rest between repetitions was 20 seconds. The lactate was measured every four repetitions. Despite the fact that in the second training session the triathlete performed four repetitions more, a lower lactate concentration is observed. In addition, body core temperature reached was lower in the second training session (from 37.4 to 38.3°C) compared to the first (from 37.5 to 38.9°C).

The performance in the races of the 2021 season is shown in Table 4. Table 5 shows the power data of cycling segment during the Olympic distance races. It is shown the average power, the average normalized power and the number of high intensity power peaks (from 550 to 1,050 W). The average time of these power peaks was 4.2 s.

**TABLE 4:** The performance in the races during the season 2021.

Event	Time (min)				
	Swimming	T1	Cycling	T2	Running
Tokyo OG	18.33	0.63	56.15	0.55	30.7
Leeds WTS	17.9	1.16	54.36	0.31	30.46
Yokohama WTS	18.6	0.93	53.71	0.4	30.43
Tokyo OG Mixed Relay	3.83	0.78	9.78	0.35	4.78
Lisbon Mixed Relay WTS	4.08	0.65	9.85	0.43	5.53

**Event**	**Speed**				
	**Swimming**	**T1**	**Cycling**	**T2**	**Running**
Tokyo OG	1.36		42.74		19.54
Leeds WTS	1.4		44.15		19.7
Yokohama WTS	1.34		44.68		19.72
Tokyo OG Mixed Relay	1.3		42.94		21.34
Lisbon Mixed Relay WTS	1.22		41.42		21.7

Time, minutes; Speed in swimming, meter per second; Speed in cycling and running, kilometer per hour. OG, olympic games; WTS, world triathlon series.

**TABLE 5:** The power profile in the cycling segment of the races during the season 2021.

Event	Avg P	Avg NP	Rep 550–1050 W	Segment orography
**Tokyo OG**	338	362	125	Flat and technical
**Leeds WTS**	307	368	87	Hills and technical
**Yokohama WTS**	319	347	175	Flat and technical

Avg P, watts average; Avg NP, normalized watts average; Rep, Number of repetitions between 580 and 1050 W.

## Discussion

The main goal of this research was to describe the training process of a world-class triathlete to prepare the Tokyo Olympic Games. The average of training volume carried out by the triathlete was less than 15 h, and the peak of training volume was almost 19 h. This training volume is less than the training volume reported by a female elite triathlete (more than 20 weekly average training hours) in her Olympic preparation for London 2012 (Mujika, 2014), but higher than the training volume reported by amateur long-distance triathletes (Neal et al., 2011; Muñoz et al., 2014; Esteve-Lanao et al., 2017; Selles-Perez et al., 2019). It is important to note that the training volume referred in our study is the active time and it does not include pauses and the time of the strength training. Compared with other endurance sports, Rønnestad and Hansen (2018) reported around 12 h of average weekly training for an elite cyclist. Kenneally et al. (2021) reported around 9 h (140 km weekly) of weekly training volume for a case of study of a world-class 5000 m athlete. As a consequence, the triathlete reported more total training volume (weekly hours) than in other cases of study about endurance athletes of other sport disciplines. Despite the fact that the volume per discipline is less than the specialists in each sport, triathletes must develop the performance of three sport modalities. Therefore, a greater number of weekly training hours would be necessary.

There are not too many studies which have analyzed the training load developed by high performance endurance athletes. TRIMPS (training impulse) have been a method to report training load in different endurance sports such as running (Esteve-Lanao et al., 2007; Stellingwerff, 2012; Muñoz et al., 2013) or cycling (Earnest et al., 2004; Rodríguez-Marroyo et al., 2011). It has also been used occasionally in triathlon (Neal et al., 2011). However, this method does not differentiate between sports disciplines with its consequent energy cost or muscle damage (Cejuela and Esteve-Lanao, 2011). It is also based on the triphasic model of training zones (Skinner and McLellan, 1980), not considering that the time to maintain the intensity of the exercise decrease exponentially from the second ventilatory threshold (Billat et al., 2012). Thus, the ECO-method seems to be more specific to quantify the training load in triathlon (Cejuela and Esteve-Lanao, 2011). Weekly average ECOs completed by the triathlete was 1,186.26 ± 320.86 ECOs. These data were in line with the ECOs reported by elite international triathletes (Olaya-Cuartero and Cejuela, 2021), but higher than those reported by elite national triathletes (Saugy et al., 2016), amateur long-distance triathletes (Esteve-Lanao et al., 2017; Selles-Perez et al., 2019) or amateur marathon runners (Esteve-Lanao et al., 2017).

TID followed by this triathlete was mainly polarized. Several previous studies have shown the effectiveness of polarized TID in elite endurance athletes (Billat et al., 2001; Fiskerstrand and Seiler, 2004; Stöggl and Sperlich, 2014, 2015). It is also observed a pyramidal TID in the first part of the season. Some other cases of study have found this TID organization over the season, involving an evolution from a more pyramidal TID during the preparatory period to a more polarized TID during the competitive period (Tjelta, 2019; Kenneally et al., 2021). Both TID models (polarized and pyramidal), present the emphasis in zone 1 (≈80%), being the remaining ≈20% of training volume distributed mainly in zone 3 in polarized TID or mainly in zone 2 in pyramidal TID (Stöggl and Sperlich, 2015). There is no clear consensus on which of these models has greater effects on performance, since few experimental studies have been carried out in this regard. Treff et al. (2017) did not report significantly differences between polarized and pyramidal training groups in elite rowers. Furthermore, Selles-Perez et al. (2019) observed how both TID were effective to improve the performance in amateur long-distance triathletes, but no clear differences between groups were found.

TLD has not been analyzed in previous studies. In this sense, even though most of the training was performed in zone 1, the triathlete completed several training weeks with more than 50% of training load in zone 3. Therefore, the analysis of training load distribution could be a new variable to be incorporated in future research, since it can provide more information about the intensity performed by endurance athletes. A ratio of 50% of training load in Zone 1 and 50% in zones two and three could represent a general guideline. Besides, the triathlete was exposed to stressful training situations such as hypoxia and heat (Racinais et al., 2015a; Flaherty et al., 2016). Thus, the weeks of training camps in altitude and the training sessions to acclimation to the Tokyo’s weather represented an added stress that must be considered.

The current consensus recommendations on training and competing in the heat (Racinais et al., 2015a) was followed to design the heat adaptation protocol. Heat acclimatization improves thermal comfort and submaximal as well as maximal exercise performance in warm-hot conditions (Racinais et al., 2015b). The first part of the acclimatization consisted on sauna baths post-exercise. Previous studies have shown the effectiveness of sauna bathing on heat acclimation (Kissling et al., 2020; Kirby et al., 2021). On the other hand, 8 weeks of progressive heat acclimatization in natural environment was performed by the triathlete previous the week of competition in Olympic Games. Training sessions with heat progressed in intensity (from low intensity training sessions to simulated competition training sessions) during these weeks, as well as the weather conditions (temperature and humidity) were harder progressively. As it is recommended in previous research, the main acclimatization block was performed the 2 weeks prior to travel, with 10 days of re-acclimation after arrival to Tokyo (Racinais et al., 2019).

Training camps at high performance center in altitude had a duration of 4 weeks. This period of time seems to be optimal to inducing accelerated erythropoiesis whereas 18 days are long enough for beneficial changes in economy or muscle buffering capacity (Millet et al., 2010). Besides, the altitude training camps were developed at an altitude of 2340 m which is defined as the optimal altitude to living high and training high (2,200–2,500) (Millet et al., 2010).

According to physiological measurements, an improvement in the triathlete’s performance is observed in the three disciplines. Relative peak ⩒O_2_ value reported by the triathlete in the second test was higher than the data reported about other elite endurance athletes such as rowers (Treff et al., 2017), swimmers (Fernandes et al., 2008), cyclists (Rønnestad and Hansen, 2018) or runners (Balsalobre-Fernández et al., 2018), and similar to other elite triathletes (Millet et al., 2003). Only some studies with top-class endurance athletes have shown similar values of VO_2max_ (Rønnestad and Hansen, 2018; Jones et al., 2021). The improvement observed in peak ⩒O_2_ was in line with the case study reported by Rønnestad and Hansen (2018), where a world-class elite cyclist had an improvement of 17% after 58 training weeks using a block periodization. However, peak ⩒O_2_ improvements in both cycling and running are much higher than other changes in ⩒O_2max_ reported in elite and well-trained athletes after a training period (García-Pallarés et al., 2010; Stoøren et al., 2012; Rønnestad et al., 2014). The triathlete performed the first test after transition period and after the first weeks of the season, where the training sessions were mainly at low intensity. So, the detraining of high intensity training zones should be considered to interpret these improvements. After the first tests, a greater amount of high intensity training sessions were prescribed in the three disciplines, which had a greater impact on the triathlete’s peak ⩒O_2_. Additionally, the decrease in total body mass (kg) and fat mass must be considered to read into the large change observed in peak ⩒O_2_ (Bassett and Howley, 2000; Mooses and Hackney, 2017) Both absolute and relative mechanical peak power output, as well as mechanical power output at ventilatory thresholds during cycling test were similar than the values reported for a world-class cyclist (Rønnestad and Hansen, 2018). It is important to know that in the cycling segment of the WTS and OG, many high power peaks are demanded, conditioned by the orography and the tactics of the race. Finally, the peak speed reported and ventilatory threshold during running test were also in line with the data reported by other elite running athletes (Ingham et al., 2012; Kenneally et al., 2021) and with the performance obtained in the races.

Finally, some limitations should be considered. Tests were only performed at the beginning and in the middle of the season. Therefore, the physiological changes produced from the middle to the end of the season are not shown. The competitions, as well as the dynamics of specific training sessions for Tokyo Olympic Games, did not allow a new test week to be scheduled at the end of the macrocycle. In addition, the manuscript shows a case study on a training macrocycle of a world-class triathlete, so the training program and the results obtained cannot be extrapolated to another type of population.

Despite these possible limitations, this research may be interesting for coaches and researches because helps to know the training characteristics of a world-class triathlete in his preparation for one of the main world sporting events. Future research with endurance athletes of this level of performance is needed.

## Data Availability

The raw data supporting the conclusion of this article will be made available by the authors, without undue reservation.
